# ETX2Vec: a fraud detection algorithm for ethereum based on temporal biased random walk strategy

**DOI:** 10.1038/s41598-026-43153-z

**Published:** 2026-04-09

**Authors:** Jiarong Lu, Bin Liao, Yi Liu, Lei Zhong

**Affiliations:** 1 College of Statistics and Data Science, Xinjiang University of Finance and Economics, Urumqi, 830012 PR China; 2 College of Big Data Statistics, Guizhou University of Finance and Economics, Guiyang, 550025 PR China; 3 School of Public Health, Xinjiang Medical University, Urumqi, 830017 PR China; 4 Xinjiang Research Center for Socio-Economic Statistics and Big Data Applications, Urumqi, China

**Keywords:** Graph embedding, Random walk, Ethernet transaction network, Fraud detection, Engineering, Mathematics and computing, Physics

## Abstract

Against the complex characteristics of the Ethereum transaction network and the limitations of existing graph embedding methods based on random walks, which fail to effectively capture transaction temporal dynamics and the flow of funds, we propose a fraud detection algorithm for Ethereum, ETX2Vec (Ethereum Transactions (TX) to Vector), which improves upon transaction subgraph construction and random walk strategies. First, in terms of transaction subgraph construction, we extract the first-order predecessor and successor neighboring nodes of the target node to reconstruct the transaction subgraph, enabling the random walk to effectively capture the complete flow of funds. Second, in the design of the random walk strategy, we introduce two key improvements: **(1)** the next node is selected based on the non-decreasing principle of transaction timestamps, effectively capturing the temporal dynamics of transactions within the network, and **(2)** a biased random walk strategy is designed based on both transaction timestamps and amounts, with a parameter $\alpha$ introduced to control the weighting of these factors when calculating transition probabilities. Experimental results show that ETX2Vec achieves an average performance of 96.04% in downstream node classification tasks, outperforming the best model in similar studies by 3.74%, and even surpassing neural network models such as GAT and GCN. This demonstrates that ETX2Vec is more effective at understanding and processing the Ethereum transaction network, leading to the learning of high-quality node embedding vectors.

## Introduction

Graph data, with its complex structure and diverse attribute types, has been widely applied to represent various real-world business scenarios. Particularly, when the relational information in the real world is abstracted and transformed into graph data, higher-order functions can be realized based on this representation. For instance, predicting potential friend relationships in social networks to recommend new connections for users^1^, constructing road networks and traffic flow networks for optimizing traffic management and scheduling^2^, analyzing the interactions between proteins and drug molecules to predict drug side effects^3^, and modeling the lending relationships between financial institutions to assess systemic risks^4^.

To fully leverage the advantages of graph data, an efficient graph data representation method is essential. Graph embedding, also referred to as network embedding or graph representation learning, aims to map the topological structure and node attributes of a graph into a low-dimensional dense vector space, thereby enabling machine learning models to process non-Euclidean data^5^. Based on the different methods of embedding nodes, existing graph embedding techniques can be primarily categorized into three types: matrix factorization-based, random walk-based, and deep learning-based methods^6^. Matrix factorization methods represent the connections between nodes in the form of a matrix and perform matrix factorization to obtain node embedding vectors. Common matrices used to represent connections include adjacency matrices, Laplacian matrices, node transition probability matrices, and Katz similarity matrices. Random walk-based methods, inspired by Word2Vec in natural language processing for word embedding, perform random walks on network nodes to generate fixed-length node sequences, capturing the network’s topological structure. These node sequences are then treated as sentences, and algorithms such as Skip-Gram are used to learn node vector representations. Deep learning-based methods typically combine techniques such as Graph Convolutional Networks (GCNs), Graph Attention Networks (GATs), and Graph Autoencoders (GAEs), integrating graph embedding with downstream task training to enable end-to-end learning. In recent years, with the deepening of research, graph embedding and learning techniques have been increasingly extended to a wide range of networked and security-related scenarios—including distributed learning systems, mobile and cloud security, and large-scale network behavior analysis—thereby greatly enriching the application scope of graph representation learning^7–12^.

The Ethereum transaction network is a typical graph-structured system, where each node represents a transaction account (address) and each edge corresponds to a specific transaction. This structural property makes graph embedding methods a natural and widely adopted choice for Ethereum fraud detection research^13–15^. However, compared with other cryptocurrency networks such as Bitcoin, the Ethereum transaction network exhibits several distinctive characteristics that substantially increase the difficulty of fraud detection. Specifically, Ethereum adopts an account-based model and supports smart contract execution, resulting in highly heterogeneous transaction behaviors, frequent bidirectional interactions between accounts, and complex multi-step fund transfer patterns. In addition, both nodes and edges in the Ethereum transaction network carry rich attribute information, including transaction timestamps, transferred amounts, and execution contexts, which play a critical role in distinguishing benign activities from fraudulent behaviors. These properties imply that fraudulent behaviors on Ethereum are often manifested not merely through static topological patterns, but through temporally ordered, direction-sensitive, and amount-dependent transaction paths. Consequently, graph embedding methods that ignore transaction attributes are insufficient to capture such dynamics. Designing embedding strategies tailored to the Ethereum transaction network is therefore essential for learning discriminative node representations.

In this work, we focus on random-walk-based graph embedding methods to model the Ethereum transaction network. Such methods typically consist of three stages: generating node sequences via random walks, learning node embeddings using the Skip-Gram model, and evaluating performance on downstream tasks such as node classification or link prediction. Among these stages, the random walk process is fundamental to embedding quality. Traditional methods, such as DeepWalk and Node2Vec, primarily focus on network topology during random walks and overlook critical transaction attributes, including timestamps and amounts. In cryptocurrency transaction networks, edges carry rich attributes such as transaction timestamps and amounts. Random-walk-based methods can effectively capture these node and edge attributes along the generated paths, allowing the walk sequences to reflect fund flow paths and temporal dynamics. Compared with other potential approaches, such as GNN-based methods, random-walk-based embedding offers a favorable balance between efficiency, scalability, and the ability to incorporate temporal and fund-flow information. By integrating attribute-aware biased transition strategies, the generated node sequences can thus better preserve the temporal, structural, and fund-flow characteristics of the Ethereum transaction network, resulting in higher-quality node embedding vectors.

Through the above analysis, combined with the review of existing research in Sect.  2 and the discussion on the complex characteristics of the Ethereum transaction network, we identify several issues in previous studies: the lack of consideration for predecessor neighboring nodes during subgraph sampling, the inability of existing biased random walk strategies to capture transaction temporal dynamics and the flow of funds, which negatively affect the quality of node embedding learning and, consequently, the performance of downstream node classification tasks. Against this background, this paper proposes a fraud detection algorithm for Ethereum, ETX2Vec, based on a temporal biased random walk strategy.

The rest of this study is organized as follows. Section  2 reviews related research; Sect.  3 presents the construction process of the ETX2Vec algorithm and the design principles of the temporal biased random walk strategy; Sect.  4 demonstrates the superiority of ETX2Vec through comparative experiments; Sect.  5 analyzes the sensitivity of the model’s key parameters; and Sect.  6 concludes the paper and provides an outlook on future work.

## Related work

### Random walk-based graph embedding methods

Random walk refers to simulating the connection paths (such as movement, transmission, interaction, or transaction) between nodes in a graph, so that the generated node sequences can map the topological structure and node attributes within the graph. Inspired by natural language processing, these node sequences are treated as sentences and input into Word2Vec to obtain low-dimensional embedding vectors for the nodes (or words), thus enabling higher-order tasks such as node classification and link prediction.

In 2014, Perozzi et al.^16^ introduced the DeepWalk algorithm, which generates node sequences through random walks on the graph and, using the Skip-Gram algorithm, achieved efficient embedding for large-scale graphs for the first time. In 2016, Grover and Leskovec^17^ proposed the Node2Vec algorithm based on DeepWalk, introducing two parameters to balance between breadth-first search (BFS) and depth-first search (DFS), and designed a flexible random walk strategy tailored to the characteristics of different networks. With the development of big data technologies, many real-world business scenarios have been abstracted into complex and diverse network structures, prompting researchers to gradually explore random walk strategies adapted to the characteristics of different networks^18,19^. Chen et al.^20^ proposed a semi-local random walk strategy to obtain the most similar node sequences, improving the link prediction task in social networks. Liu et al.^21^ proposed the Node2Vec+ model for gene interaction networks, which fully considers edge weights when calculating transition probabilities, and demonstrated that Node2Vec + is more resistant to noise in downstream disease prediction tasks. Berahmand et al.^22^ introduced an improved local random walk strategy for complex networks, which uses biases to guide each step of the random walk toward more influential nodes. Experimental results show that this strategy outperforms traditional random walk methods in downstream link prediction tasks.

Compared to matrix factorization and deep learning-based methods, random walk-based graph embedding methods can adjust the random walk strategy according to the characteristics of different networks. This allows for more flexible exploration of both local and global structural information of the graph, while offering a degree of interpretability and high computational efficiency. Therefore, they are an important tool for handling complex networks (such as Ethereum transaction networks) and large-scale networks.

### Random walk-based ethereum fraud detection research

With the rapid growth of the Ethereum ecosystem, various security threats and fraudulent activities have also increased. Illicit activities such as double spending attacks, phishing scams, and smart contract exploits have become more frequent, severely damaging user interests and impacting the healthy development of the Ethereum ecosystem^23,24^. The rapid advancement of artificial intelligence, big data, and machine learning technologies has provided solutions for Ethereum fraud detection. Early fraud detection methods primarily relied on manually constructed features, such as transaction amounts, frequencies, and time intervals^25–28^. Machine learning techniques were used to train fraud detection models that could identify abnormal transaction behaviors to some extent. However, manually constructing features often requires significant human intervention and struggles to capture complex transaction patterns, making it less effective in the face of the complex and highly dynamic blockchain environment.

In recent years, graph embedding methods have gradually become the mainstream approach in Ethereum fraud detection research. Specifically, random walk-based methods have inherent advantages in extracting key information such as transaction temporal dynamics and the flow of funds. The differences in current research mainly lie in two aspects: the construction of transaction subgraphs and the design of random walk strategies. The construction of transaction subgraphs mainly aims to address model computational efficiency, while the design of random walk strategies aims to more effectively extract the complex characteristics of the transaction network. First, in terms of transaction subgraph construction, Yuan et al.^29^ constructed transaction subgraphs by extracting the first-order successor neighbors of the central node, and performed embedding learning and node classification using Node2Vec and SVM, respectively. Xia et al.^30^ constructed K-hop directed ego-graphs for nodes and used the Graph2Vec algorithm to learn node embedding vectors, followed by training a decision tree for downstream node classification tasks. Secondly, in terms of random walk strategies, Wu et al.^31^ proposed the trans2vec model, which for the first time designed a biased random walk strategy based on transaction attributes. They demonstrated that this strategy outperformed traditional random walk strategies (such as DeepWalk and Node2Vec) in downstream node classification tasks. Lin et al.^32^, building upon the strategy proposed in [31], trained a link prediction model on the Ethereum transaction network and further confirmed that the biased random walk strategy, which considers transaction attributes, more effectively captures the network’s topological structure and transaction attributes. As a result, this strategy has been widely adopted in subsequent research to understand and process cryptocurrency transaction networks^33,34^.

Designing a reasonable random walk strategy is key to obtaining high-quality node embedding vectors. Before that, let’s first discuss the unique characteristics of the Ethereum transaction network and the limitations of existing research.

Unlike traditional social networks or information dissemination networks, cryptocurrency transaction networks have the following distinct features: (1) nodes and edges in cryptocurrency transaction networks carry rich attribute information, such as transaction timestamps, amounts, and the occurrence of multiple or bidirectional transactions between nodes; (2) the connections between nodes in the Ethereum transaction network inherently contain potential information such as transaction temporal dynamics and the flow of funds; (3) information about recent and large transactions between nodes and their predecessor and successor neighboring nodes is crucial for distinguishing node categories.

Given these differences, existing research still has the following limitations when dealing with the Ethereum transaction network: (1) traditional random walk-based graph embedding methods (such as DeepWalk, Node2Vec, etc.) cannot extract network attributes like transaction timestamps and amounts; (2) existing biased random walk strategies fail to effectively capture transaction temporal dynamics and the flow of funds within the network; (3) existing studies in constructing transaction subgraphs only extract the successor neighboring nodes of the target node, lacking the extraction of information such as the source path of funds.

To address this issue, this paper proposes ETX2Vec, a fraud detection method for Ethereum based on a temporal-biased random walk strategy. The core contribution of ETX2Vec lies in systematically integrating transaction directionality and temporal information into the random walk process, which is essential for modeling fund-flow behaviors in Ethereum transaction networks. The main design components are summarized as follows:

#### Improved transaction subgraph construction method

The Ethereum transaction subgraph is constructed by extracting the first-order predecessor and successor neighboring nodes of the target node. This method not only highly restores the structural information of the transaction subgraph in the original graph but also fully extracts the target node’s fund inflow and outflow records, enabling the random walk to effectively capture the complete flow of funds.

#### Improved random walk strategy

On one hand, the strategy selects the next node based on the non-decreasing principle of transaction timestamps, ensuring that the generated node sequence reflects the transaction temporal dynamics of nodes in the network. On the other hand, a biased random walk strategy is designed based on transaction timestamps and amounts, with a parameter $\alpha$ introduced to adjust the weights of these factors in the node transition probability calculation. This allows the generated node sequence to effectively capture transaction temporal dynamics and large-scale fund flow paths.

## Framework and method of ETX2Vec

**Fig. 1:**
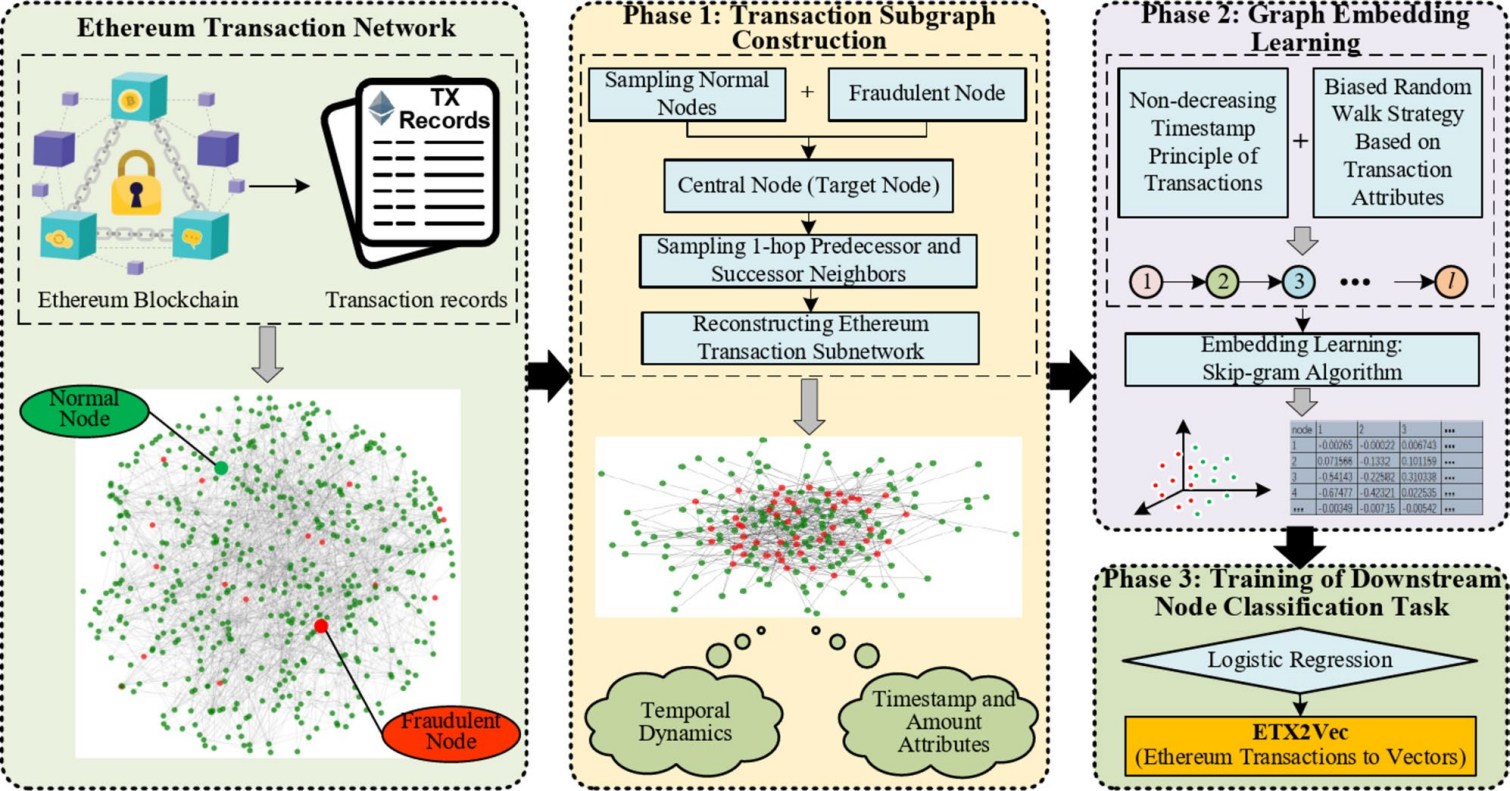
ETX2Vec Model Construction Process.

This section presents the design principles and construction process of the ETX2Vec algorithm, which consists of three main stages: Ethereum transaction subgraph construction, graph embedding learning based on a temporal biased random walk strategy, and training of downstream node classification tasks. The overall technical framework of the algorithm is illustrated in Fig. 1. The definition of the Ethereum transaction network is as follows:

### Definition 1

**Ethereum Transaction Network** $\mathbb{G}$

Ethereum transaction records can be abstracted into graph data^35^. Given a set of Ethereum addresses within a specific time frame, a directed multigraph $\mathbb{G}=(V,E,Y)$ is constructed based on the flow of funds between addresses, transaction amounts, and transaction timestamps. Let the total number of nodes in the transaction network be $n$, i.e., $\left|V\right|=n$, then the set of nodes $V={\{v}_{1},{v}_{1},\cdots{v}_{n}\}$ represents all the transaction addresses within that time period. Let the total number of edges in the transaction network be $m$, i.e., $\left|E\right|=m$. For each ${e}_{ij}\in E$, where $i,j=\mathrm{1,2},\cdots n,i\ne j$, define ${e}_{ij}=\left\{\right({v}_{i},{v}_{j},{t}_{ij},{a}_{ij}\left)\right\}$, which represents a transaction where address ${v}_{i}$ transfers an amount ${a}_{ij}$ of Ether to address ${v}_{j}$ at timestamp ${t}_{ij}$. Here, ${v}_{i},{v}_{j}\in V,{t}_{ij}\in{Z}^{+}$ represents the timestamp attribute of the edge, and ${a}_{ij}\in{R}^{+}\cap\left\{0\right\}$ represents the transaction amount attribute of the edge.

### Transaction subgraph construction

Processing large-scale transaction networks with over 2 million node addresses is highly time-consuming [29]. Therefore, it is necessary to construct transaction subgraphs as input for graph embedding learning. Given that the number of normal nodes in the transaction network is much larger than the number of fraud nodes, and constructing the transaction subgraph may lead to the loss of information from the original graph, this could cause overfitting and poor generalization ability during downstream classification task training. To address this, this paper constructs Ethereum transaction subgraphs based on the 1-hop predecessor and successor neighbor nodes of the target node. The construction process is divided into the following four steps:

(1) Remove isolated nodes that are disconnected from the original transaction network, nodes with low frequency, and invalid edges where the transaction amount is zero, in order to reduce noise and unnecessary computational load, thus optimizing the structure of the transaction network.

(2) To balance the proportion of node categories, randomly select a number of normal nodes equal to the number of fraud nodes, and combine them with all fraud nodes to form the target node set. Let the number of fraud nodes in the original transaction network be denoted as s. Then, the target node set is represented as ${\mathbb{C}}_{sub}=\left\{{v}_{1},{v}_{2},\cdots,{v}_{2s}\right\}$, where $\left|{\mathbb{C}}_{sub}\right|=2s$.

**Fig. 2:**
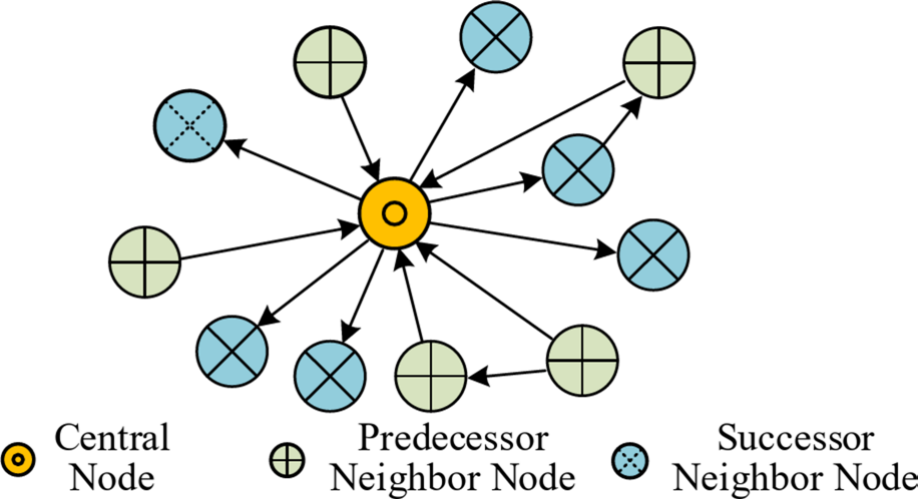
Illustration of the 1-hop predecessor and successor neighbor nodes of a central node.

(3) To improve the local similarity between the transaction subgraph and the original transaction network, the strategy of extracting the 1-hop predecessor and successor neighbor nodes of the target node to construct the transaction subgraph is adopted, as shown in Fig. 2. First, for each ${v}_{i}\in{\mathbb{C}}_{sub}$, extract all its 1-hop predecessor and successor neighbor nodes from the original transaction network, and merge them with ${\mathbb{C}}_{sub}$ to form the node subset ${V}_{sub}$, where ${V}_{sub}\subset V$ and $\left|{V}_{sub}\right|<\left|V\right|=n$. Next, for any pair of nodes ${v}_{i},{v}_{j}\in{V}_{sub},i\ne j$, if there exists a transaction edge ${e}_{ij}$in the original transaction network $G$, we include ${e}_{ij}$to form the edge subset ${E}_{sub}$. Finally, the Ethereum transaction subgraph is reconstructed as ${G}_{sub}=({V}_{sub},{E}_{sub})$.

Compared with existing methods that only consider K-hop successor neighbors of the central node^29,30^, the proposed strategy explicitly incorporates both predecessor and successor nodes, enabling the subgraph to preserve complete incoming and outgoing fund flows of the target account. This bidirectional construction provides a more faithful local approximation of the original transaction network and establishes a consistent structural basis for the subsequent time-aware random walk process.

Restricting the neighborhood scope to 1-hop predecessor and successor nodes is a deliberate design choice that balances modeling expressiveness and computational efficiency. Direct neighbors capture the most informative transactional interactions, corresponding to immediate inflow and outflow behaviors that are particularly effective for identifying common fraud patterns such as phishing and direct scams. In contrast, higher-order neighborhoods may contain long-range transaction paths relevant to complex laundering behaviors, but they also introduce a rapidly increasing number of indirect and weakly related paths. From a computational perspective, blockchain transaction networks often exhibit high degree variability. Expanding the neighborhood to $k$-hop nodes can lead to exponential growth in subgraph size in the worst case, on the order of $O\left({d}^{k}\right)$, where $d$denotes the average node degree. Such expansion substantially increases the cost of subgraph construction and random walk sampling, and enlarges the walk state space, thereby reducing the effectiveness of temporal and amount-based transition biases. In contrast, the 1-hop bidirectional neighborhood constrains the subgraph size to $O\left(d\right)$, ensuring scalability to large-scale Ethereum transaction graphs. Moreover, excessively large neighborhoods may introduce significant path randomness, diluting temporal consistency along longer transaction paths and potentially increasing false negatives for complex fraud detection. By focusing on 1-hop predecessor–successor structures, the proposed method favors stable and semantically coherent fund-flow patterns, which enhances the robustness, interpretability, and discriminative power of the learned node embeddings.

### Graph Embedding Learning

This section introduces the design principles of the temporal biased random walk strategy. Specifically, the strategy selects the next node based on the non-decreasing timestamp principle, and considers three sampling strategies when selecting the next node: timestamp-biased, amount-biased, and a balance between timestamp and amount biases.

#### Definition 2

**Temporal Random Walk Strategy (TRW)**

Let ${v}_{i-1}$ and ${v}_{i}$ be the previous and current nodes in the node sequence, where $T({v}_{i-1},{v}_{i})$ represents the timestamp of the transaction from ${v}_{i-1}$ to ${v}_{i}$. Let the set of $q$ first-order successor neighbors of node ${v}_{i}$ be denoted as ${V}_{nbrs}^{\left(i\right)}$, then the next node set ${V}_{temp-nbrs}^{\left(i\right)}$, which satisfies the non-decreasing timestamp principle, is defined as follows:

1 $${V}_{temp-nbrs}^{\left(i\right)}=\left\{{v}_{i+1}\right|{v}_{i+1}\in{V}_{nbrs}^{\left(i\right)},T({v}_{i},{v}_{i+1})>T({v}_{i-1},{v}_{i})\}$$

where $\left|{V}_{temp-nbrs}^{\left(i\right)}\right|=h$ and $\left|{V}_{nbrs}^{\left(i\right)}\right|=q$, with $h\leq$.

**Fig. 3:**
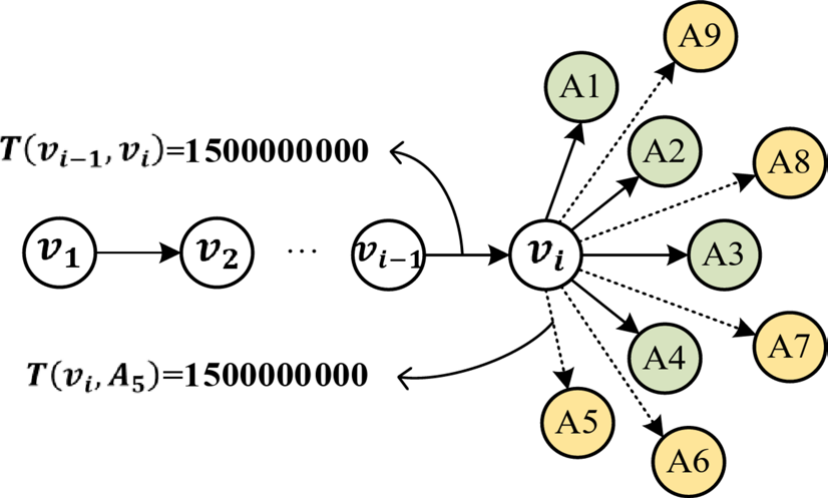
Illustration of the Non-decreasing Timestamp Principle.

As shown in Fig. 3, in the node sequence, $T\left({v}_{i-1},{v}_{i}\right)=1500000000$, and ${V}_{nbrs}^{\left(i\right)}=\{{A}_{1},{A}_{2},\cdots,{A}_{9}\}$ is the set of successor neighbor nodes of ${v}_{i}$, arranged in ascending order based on the transaction timestamps with $T\left({v}_{i},{A}_{5}\right)=1500000000$, the next node set ${V}_{temp-nbrs}^{\left(i\right)}=\{{A}_{5},{A}_{6},\cdots{A}_{10}\}$ satisfies the condition in Definition 2. The temporal random walk strategy ensures that the generated node sequence exhibits non-decreasing time, but it is still insufficient to capture features like recent and large transactions in the network. Therefore, this paper fully considers the influence of transaction timestamps and amounts on the node transition probabilities and designs three different biased random walk strategies.

Considering that transaction attributes in Ethereum networks exhibit highly heterogeneous numerical scales, directly incorporating raw values into transition probability computation may lead to severe imbalance among different factors. In particular, transaction timestamps often span long temporal ranges and possess significantly larger magnitudes than other attributes, while transaction amounts typically follow a long-tail distribution. If used without appropriate normalization, such disparities may cause certain attributes to dominate the random walk transition process, thereby weakening the contribution of other informative features and reducing both numerical stability and interpretability.

To address this issue, we adopt a linear normalization strategy to remap transaction attribute values into a bounded range while preserving their relative ordering and monotonic trends. This design allows different attributes to contribute to transition probability computation in a controlled and comparable manner, without altering their inherent semantic relationships. Notably, the purpose of normalization in ETX2Vec is not to retain exact proportional differences between attribute values, but to ensure stable probabilistic modeling and meaningful integration of heterogeneous transaction information within the random walk framework.

Formally, let $x$ denote the original value of a transaction attribute, and let ${x}_{\mathrm{m}\mathrm{i}\mathrm{n}}$ and ${x}_{\mathrm{m}\mathrm{a}\mathrm{x}}$ represent its minimum and maximum values in the transaction network, respectively. The normalized value ${x}^{{\prime}}$ is computed as:

2 $${x}^{{\prime}}=\frac{x-{x}_{min}}{{x}_{max}-{x}_{min}}\cdot{X}_{sup}+{X}_{inf}$$

where ${X}_{sup}$ and ${X}_{inf}$ be the upper and lower bounds of the preset mapping range.

#### Definition 3

**Biased Random Walk Strategy**

#### Temporal Unbiased Random Walk Strategy (TURW)

Let ${v}_{i}$ be the current node in the node sequence. Then, for ${v}_{i}$, each neighbor node ${v}_{i+1}^{\left(j\right)}$ selected as the next node has an equal probability of being chosen, according to Definition 2:

3 $${P}_{0}^{\left(j\right)}=\frac{1}{\left|{V}_{temp-nbrs}^{\left(i\right)}\right|}$$

where $${v}_{i+1}^{\left(j\right)}\in{V}_{temp-nbrs}^{\left(i\right)},j=1,2,\cdots h,\left|{V}_{temp-nbrs}^{\left(i\right)}\right|=h$$.

#### Temporal & Timestamp Biased Random Walk Strategy (TTBRW)

Let ${v}_{i-1},{v}_{i}$ be the previous node and the current node in the node sequence, and $T({v}_{i-1},{v}_{i})$ represent the transaction timestamp between them. Let ${V}_{temp-nbrs}^{\left(i\right)}=\{{v}_{i+1}^{\left(1\right)},{v}_{i+1}^{\left(2\right)},\cdots,{v}_{i+1}^{\left(h\right)}\}$ be the set of $h$ ${v}_{i}$ satisfying Definition 2, and $T({v}_{i},{v}_{i+1}^{\left(j\right)})$ represent the transaction timestamp between ${v}_{i}$ and ${v}_{i+1}^{\left(j\right)}$. Let ${\varDelta t}_{j}=T\left({v}_{i},{v}_{i+1}^{\left(j\right)}\right)-T({v}_{i-1},{v}_{i})$ represent the time interval between the previous transaction (funds flowing into ${v}_{i}$) and the next transaction (funds flowing out of ${v}_{i}$), then the probability of selecting each neighbor node ${v}_{i+1}^{\left(j\right)}$ as the next node for ${v}_{i}$, satisfying Definition 2, is:

4 $${P}_{t}^{\left(j\right)}=\frac{{t}_{sup}-{\varDelta t}_{j}}{{\sum}_{j=1}^{h}({t}_{sup}-{\varDelta t}_{j})}$$

where ${t}_{sup}$ denotes the upper bound of the normalized mapping for the transaction timestamp $t$. This strategy prioritizes the selection of neighboring nodes whose transaction timestamps are closer to that of the previous node, meaning that the smaller the ${\varDelta t}_{j}$, the higher the likelihood of choosing that neighbor as the next node. Consequently, the generated node sequence effectively captures the temporal dynamics of transactions and the flow paths of funds.

#### Temporal & Amount Biased Random Walk Strategy (TABRW)

Let ${v}_{i}$ be the current node in the node sequence, and ${V}_{temp-nbrs}^{\left(i\right)}=\{{v}_{i+1}^{\left(1\right)},{v}_{i+1}^{\left(2\right)},\cdots,{v}_{i+1}^{\left(h\right)}\}$ represent the set of $h$ neighboring nodes of ${v}_{i}$ satisfying the conditions in Definition 2. Let $A({v}_{i},{v}_{i+1}^{\left(j\right)})$ be the transaction amount between ${v}_{i}$ and ${v}_{i+1}^{\left(j\right)}$. Then, the probability of selecting each neighboring node ${v}_{i+1}^{\left(j\right)}$ as the next node in the sequence is defined as:

5 $${P}_{a}^{\left(j\right)}=\frac{A({v}_{i},{v}_{i+1}^{\left(j\right)})}{{\sum}_{j=1}^{h}A({v}_{i},{v}_{i+1}^{\left(j\right)})}$$

This strategy prioritizes selecting neighboring nodes ${v}_{i+1}^{\left(j\right)}$ with larger transaction amounts when determining the next node for ${v}_{i}$, focusing on capturing the flow paths of large transactions within the network.

#### Temporal & Timestamp and Amount Biased Random Walk Strategy (TTABRW)

To comprehensively consider the influence of both transaction timestamp and transaction amount on node selection, the node selection probabilities from (2) and (3) are combined. Thus, the probability of each neighbor node ${v}_{i+1}^{\left(j\right)}$ being selected as the next node for ${v}_{i}$, satisfying the definition 2, is given by:

6 $${P}^{\left(j\right)}=\alpha{P}_{t}^{\left(j\right)}+(1-\alpha){P}_{a}^{\left(j\right)}$$

where $0\le\alpha\le1$ is a weight parameter that controls the relative significance of the transaction timestamp and transaction amount in determining the next node. A larger value of $\alpha$ gives greater importance to the transaction timestamp in the node transition probability, while a smaller value places more emphasis on the transaction amount.

Based on the node sequences generated by the above different walk strategies, this paper adopts the Skip-Gram model to learn the embedding vectors of the center nodes. Skip-Gram is a widely used data representation learning framework, originally proposed for natural language processing^36^. By training on large-scale textual data, it maps words to a low-dimensional vector space, such that semantically similar words are located close to each other in the vector space. In this paper, node sequences are treated as “sentences” and nodes as “words”. The Skip-Gram model learns the vector representation of each node by maximizing the co-occurrence probability between the center word (node) and its context words (neighboring nodes). In the Skip-Gram model, the vector representation of each node is learned by optimizing the following objective function using stochastic gradient descent:

7 $$\underset{f}{{max}}\sum_{v\in V}\mathrm{log}Pr\left({N}_{S}\right(v\left)\right|f\left(v\right))$$

The training process of the ETX2Vec model is shown in Algorithm 1. The input to the algorithm includes the Ethereum transaction subgraph ${\mathbb{G}}_{sub}$, a subset of center nodes ${\mathbb{C}}_{sub}$, the four temporal random walk strategies, and key parameters. The output is the node embedding vector matrix $M$. In line 1, the transaction subgraph ${\mathbb{G}}_{sub}$ is read, including node labels and transaction timestamp and amount attributes. Line 2 defines the initialization of the node sequence, used to store the walked nodes in real-time. Line 3 defines the different temporal biased random walk strategies. From line 4, node sequences are generated under different strategies, with multiple random walks performed for each node, controlled by $r$. In lines 5 to 9, the center node undergoes a random walk to obtain the second node. Since the starting node has no previous node, temporal constraints are not considered in this step. Line 10 retrieves all neighboring nodes of the second node. In lines 11 to 15, the neighbor nodes satisfying the temporal non-decreasing principle are selected, based on definition 3. In lines 16 to 17, node sequences of length $l$ for the center node are generated using different random walk strategies. Line 18 iteratively obtains the node sequence for each center node. Lines 19 to 20 gather $r$ node sequences for each center node. Finally, in lines 21 to 22, the node sequences are fed into the Skip-gram model, which learns the center node’s embedding vector matrix $M$, serving as input for the downstream node classification task.

**Figure:**
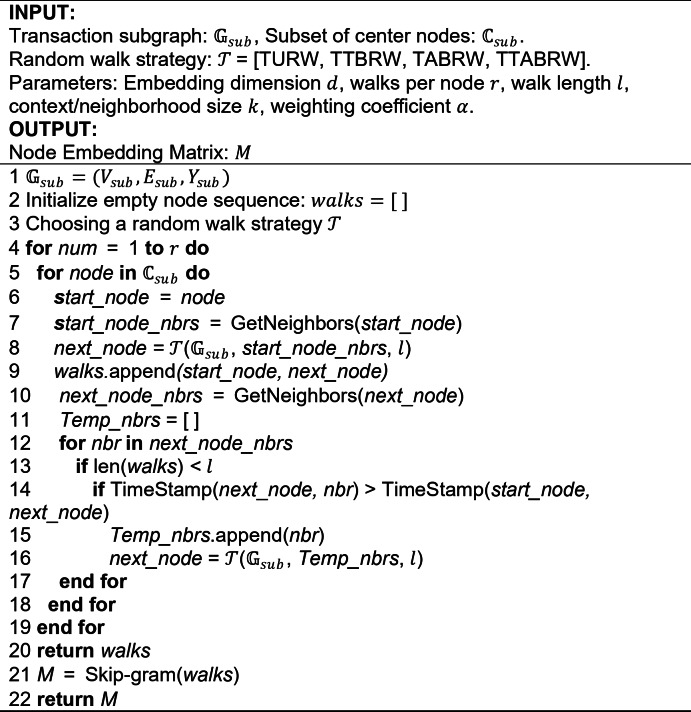
**Algorithm 1**: The training process of the ETX2Vec.

It should be noted that ETX2Vec adopts a transductive learning setting, operating on the constructed Ethereum transaction network within the studied time frame. Instead of processing the entire graph at once, the method constructs local transaction subgraphs for each target node and performs time-biased random walks on these subgraphs. This design confines computation to 1-hop predecessor and successor neighborhoods, which reduces the graph size and limits computational overhead, while still capturing the essential structural and temporal patterns required for effective node embedding. Consequently, although ETX2Vec is transductive, the local subgraph strategy ensures that training is computationally feasible even on large-scale Ethereum transaction networks.

## Experiment and results analysis

### Experimental environment and evaluation metrics

The experimental operating system is Windows 11, the CPU is an AMD Ryzen 7 7840 H with Radeon 780 M Graphics 3.80 GHz, and the memory configuration is 128GB-DDR5-5600 MHz (16GB*8). The Python version used is 3.9, networkx version is 2.8.8, scikit-learn version is 1.5.0, and all experiments were debugged on the PyCharm 2024.1.1 development platform.

This paper evaluates the performance of ETX2Vec using five classification algorithm evaluation metrics: Accuracy (Acc), Precision (Pre), Recall (Rec), F1 score (F1), and Average Performance (AVG), defined as follows:

8 $$Acc=\frac{TP+TN}{TP+FP+TN+FN}\times100\mathrm{\%}$$

9 $$Pre=\frac{TP}{TP+FP}\times100\mathrm{\%}$$

10 $$Rec=\frac{TP}{TP+FN}\times100\mathrm{\%}$$

11 $$F1=\frac{2\times Pre\times Rec}{Pre+Rec}\times100\mathrm{\%}$$

12 $$AVG=\frac{1}{4}\left(Acc+Pre+Rec+F1\right)\times100\mathrm{\%}$$

### Data

The Ethereum transaction dataset used in this paper is shared by Wu et al.^31^. This dataset consists of transaction records from a set of Ethereum addresses, including both legitimate addresses and fraudulent addresses reportedly involved in phishing, illegal fundraising, money laundering, and other fraudulent activities. Each transaction record includes the sending address, receiving address, transaction timestamp, and transaction amount. On the Ethereum blockchain, there may be multiple bidirectional transaction records between any two account addresses. Therefore, using Python’s networkx library, it is constructed into a directed multigraph $\mathbb{G}$, where each edge carries the transaction timestamp and amount.

Considering the large scale of graph $\mathbb{G}$ and the highly imbalanced distribution of fraudulent and legitimate nodes, this paper constructs the Ethereum transaction subgraph ${\mathbb{G}}_{sub}$ based on the strategy proposed in Sect.  3.2. Specifically, 1165 legitimate nodes are randomly selected from all legitimate nodes in the original graph $\mathbb{G}$, and combined with 1165 fraudulent nodes to form the target node set ${\mathbb{C}}_{sub}$. Then, for each target node, its 1-hop predecessor and successor neighbor nodes are selected and merged with the target nodes to form the node subset ${V}_{sub}$. Finally, the transaction information of all nodes in ${V}_{sub}$ is traversed in the original graph $\mathbb{G}$, and the Ethereum transaction subgraph ${\mathbb{G}}_{sub}$ is reconstructed. The statistical information for graphs $\mathbb{G}$ and ${\mathbb{G}}_{sub}$ is shown in Table 1.

**Table 1:** Ethereum Transaction Network Statistics.

Dataset	Nodes	Edges	Illicit nodes
$\mathbb{G}$	2,973,489	13,551,303	1165
${\mathbb{G}}_{sub}$	25,671	331,945	1165

### Experimental results analysis

To ensure fair and consistent experimental comparisons, all models are initialized with the following unified hyperparameters: the embedding dimension is set to $d=128$, walk length $l=20$, number of walks per node $r=10$, context window size $k=5$, return parameter $p=0.25$, and in–out parameter $q=0.75$. In addition, the temporal–amount weighting coefficient is fixed at $\alpha=0.6$, and transaction attribute values are linearly mapped into the range $[{\boldsymbol{X}}_{\boldsymbol{i}\boldsymbol{n}\boldsymbol{f}},{\boldsymbol{X}}_{\boldsymbol{s}\boldsymbol{u}\boldsymbol{p}}]=[1,1000]$.

Firstly, the constructed Ethereum transaction subgraph ${G}_{sub}$is used as the input for graph representation learning, where node sequences are generated based on different temporal biased random walk strategies. These sequences are then fed into the Skip-Gram model to learn node embedding vectors, which are subsequently divided into 80% training data and 20% testing data. Finally, a logistic regression classifier is employed to perform the downstream fraud detection task.

To comprehensively validate the effectiveness of the proposed method, we conduct three groups of comparative experiments: **Experiment 1** compares the proposed temporal biased random walk strategies with classic random-walk-based baseline methods, including DeepWalk and Node2Vec, as well as representative graph neural network models, namely GCN and GAT. **Experiment 2** further compares ETX2Vec with graph embedding methods proposed in previous studies. In addition, **Experiment 3** evaluates the robustness of the learned embeddings by comparing logistic regression with ten mainstream classifiers in the downstream classification task.

#### Experiment 1: Comparison with Baseline Methods

**Fig. 4:**
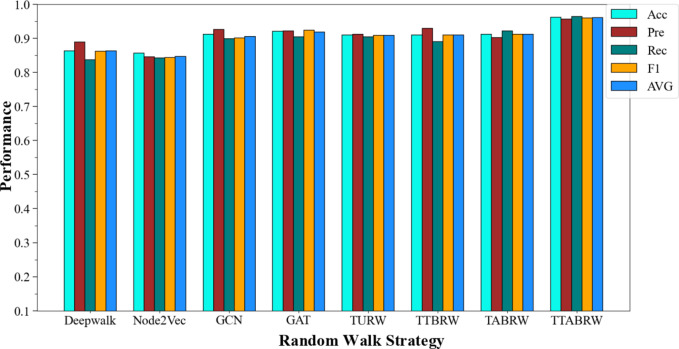
Comparison of Model Classification Performance with Different Walk Strategies.

Experiment 1 compares the four temporal biased random walk strategies designed in ETX2Vec with four baseline models, namely DeepWalk, Node2Vec, GCN, and GAT. As shown in Fig. 4 and summarized in Table 2, the proposed TTABRW strategy consistently outperforms all baseline methods in terms of accuracy, precision, recall, and F1-score.

**Table 2:** Performance Comparison of Temporal Biased Random Walk Strategies and Baseline Models.

Model	Acc / %	Pre / %	Rec / %	F1 / %	AVG / %
Deepwalk	86.27	88.89	83.68	86.21	86.26
Node2Vec	85.62	84.58	84.19	84.38	84.69
GCN	91.23	92.56	89.85	90.05	90.49
GAT	92.08	92.17	90.37	92.39	91.86
TURW	90.98	91.23	90.43	90.83	90.87
TTBRW	90.99	92.95	89.03	90.95	90.98
TABRW	91.20	90.21	92.17	91.18	91.19
**TTABRW**	**96.14**	**95.58**	**96.43**	**96.00**	**96.04**

As reported in Table 2, DeepWalk and Node2Vec achieve average performances of 86.26% and 84.69%, respectively. Although Node2Vec introduces biased walks to balance local and global structures, its transition strategy remains topology-driven and fails to incorporate transaction-specific attributes, resulting in limited effectiveness on Ethereum transaction networks. In contrast, GCN and GAT exhibit stronger performance, with average scores of 90.49% and 91.86%, respectively. This improvement is expected, as GNN-based methods can aggregate neighborhood information through learnable message-passing mechanisms. However, their performance remains constrained by fixed receptive fields and the lack of explicit modeling of transaction temporal order and fund flow directionality, which are critical for fraud detection in transaction graphs.

The four proposed strategies—TURW, TTBRW, TABRW, and TTABRW—achieve average performances of 90.87%, 90.98%, 91.19%, and 96.04%, respectively, outperforming all baseline methods, including GCN and GAT. This demonstrates that explicitly incorporating transaction timestamps and amounts into the random walk process substantially enhances embedding quality. Specifically, TURW enforces a non-decreasing timestamp constraint, leading to a performance improvement of 4.61%–6.18% over DeepWalk and Node2Vec, highlighting the importance of temporal consistency. TTBRW and TABRW further introduce biased transition probabilities based on transaction timestamps and amounts, focusing on continuous transaction dynamics and large fund flows, respectively, and achieve incremental improvements over TURW.

Notably, the TTABRW strategy, which jointly models temporal order and transaction amount with a balancing parameter $\alpha$, achieves the best overall performance, reaching an average score of 96.04% when $\alpha=0.6$. This result indicates that TTABRW is able to capture both fine-grained temporal dependencies and salient fund flow patterns, generating node sequences that more faithfully reflect real transaction behaviors. Compared with GCN and GAT, TTABRW benefits from flexible, path-based context modeling that naturally aligns with multi-step fund transfer patterns, resulting in more discriminative node embeddings for fraud detection.

Overall, the comparative experiments demonstrate that while GNN-based methods provide strong baselines, the proposed temporal biased random walk strategy—especially TTABRW—offers a more effective and scalable solution for modeling Ethereum transaction networks. Therefore, TTABRW is selected as the core random walk strategy in ETX2Vec for the subsequent experiments.

#### Experiment 2: Comparison with Similar Works

To validate the effectiveness of ETX2Vec in handling cryptocurrency transaction networks, the experiment 2 compares ETX2Vec with existing Ethereum fraud detection models based on random walk-based graph embedding methods proposed in previous works. Additionally, this section also includes a comparison with three deep learning-based graph embedding methods: Graph2Vec, PDGNN, and GTN2Vec.

**Table 3:** Performance Comparison of ETX2Vec with Existing Studies.

Model	Graph Embedding Method	Acc / %	Pre / %	Rec / %	F1 / %	AVG / %
Node2Vec^29^	Random Walk-based Method	/	/	/	84.60	84.60
trans2vec^31^		/	92.70	89.30	90.80	90.93
SIGTRAN^33^		94.20	94.40	94.00	94.20	94.20
HNRL^34^		/	88.90	/	95.70	92.30
Graph2Vec^30^	Deep Learning-based Method	/	81.32	82.71	81.99	82.01
PDGNN^37^		90.14	88.75	92.05	90.33	90.32
GTN2Vec^38^		95.70	95.30	96.40%	95.90	95.83
**ETX2Vec**		**96.14**	**95.58**	**96.43**	**96.00**	**96.04**

As shown in Table 3, a comprehensive comparison based on accuracy, precision, recall, F1 score, and average performance demonstrates that the ETX2Vec algorithm outperforms all other methods in the downstream Ethereum fraud detection task. Firstly, among the models based on traditional random walk methods, including Node2Vec^29^ and SIGTRAN^33^, Node2Vec exhibits the lowest performance. This indicates that the pure random walk strategy lacks the capability to capture the intricate characteristics of the transaction network. In contrast, SIGTRAN achieves an average performance of 94.20%, primarily because this model combines manually engineered features with the vector features obtained through graph embedding.

Next, the models that employ biased random walk strategies, such as trans2vec^31^ and HNRL^34^, achieve performance levels exceeding 90%, suggesting that these strategies are capable of effectively extracting transactional information from the network, resulting in high-quality node embeddings. When compared to these baseline algorithms, ETX2Vec demonstrates a significant improvement of 3.74% over HNRL and 5.11% over trans2vec in terms of average classification performance. This enhancement can be attributed to two key factors: (1) Transaction Subgraph Construction: In this work, transaction subgraphs are constructed by extracting the 1st-order predecessor and successor neighbors of target nodes. This not only enables the transaction subgraph to faithfully reflect the structural information of the original graph but also captures the complete inflows and outflows of funds related to the target nodes, providing a comprehensive view of the financial transaction path. (2) Time-Aware Biased Random Walk Strategy: The proposed strategy selects nodes based on the non-decreasing order of transaction timestamps, while also considering both timestamp and transaction amount in the calculation of node transition probabilities. This ensures that the generated node sequences not only capture the temporal dynamics of transactions but also effectively trace large fund flows across the network.

This highlights the intrinsic advantage of random walk-based strategies in understanding and processing Ethereum transaction networks, which exhibit complex and multifaceted characteristics. Specifically, a well-designed random walk strategy can effectively capture key features such as continuous, frequent, and large transactions that are typical of fraudulent nodes. As a result, ETX2Vec outperforms even advanced graph neural network models such as GAT and GCN in downstream node classification tasks.

#### Experiment 3: Comparison of Downstream Classifiers

Given that the choice of downstream classifiers can notably influence node classification performance, the experiment 3 evaluates the default Logistic Regression (LR) model against 10 widely-used machine learning classifiers: KNN, SVM, Decision Tree, MLP, Random Forest, Extra Trees, XGBoost, GBDT, LightGBM, and AdaBoost. For each classifier, the default settings in scikit-learn are applied. The data is split into an 8:2 training-to-testing ratio, with a fixed random_state value to ensure both fairness and reproducibility in the experiments.

**Table 4:** The comparison of different downstream classifiers.

Model	Acc / %	Pre / %	Rec / %	F1 / %	AVG / %
KNN	74.46	97.30	48.21	64.48	71.11
Decision Tree	86.05	83.54	88.39	85.90	85.97
AdaBoost	91.20	92.17	89.29	90.70	90.84
MLP	92.27	94.76	88.84	91.71	91.90
SVM	93.56	94.50	91.96	93.21	93.31
Random Forest	93.56	90.42	96.88	93.53	93.60
XGBoost	94.85	93.48	95.98	94.71	94.76
GBDT	95.49	94.71	95.98	95.34	95.38
Extra Trees	95.71	94.74	96.43	95.58	95.61
LightGBM	95.71	94.74	96.43	95.58	95.61
**LR**	**96.14**	**95.58**	**96.43**	**96.00**	**96.04**

As shown in Table 4, the Logistic Regression (LR) model used in this study outperforms other models in classification performance. Firstly, most ensemble learning classifiers perform better than individual nonlinear classifiers, likely because ensemble models can build more accurate and robust classifiers by reducing variance, increasing diversity, and avoiding overfitting during training. Secondly, LR stands out among the models for several reasons: (1) LR has a fast training speed, especially on large-scale datasets. Its simple mathematical form and optimization algorithms (such as gradient descent) enable it to complete parameter estimation in a shorter time. Moreover, LR handles high-dimensional sparse data effectively, which is particularly important for node classification tasks, especially when the embedding vectors have a uniform scale and low variance, allowing LR to make better use of these features for classification. Lastly, LR prevents overfitting through L1 or L2 regularization, ensuring superior generalization ability of the model on unseen data.

In summary, LR performs the best in the downstream node classification task, while models such as Random Forest, XGBoost, GBDT, Extra Trees, and LightGBM also demonstrate distinct advantages in various aspects. The common characteristic of these models is their ability to enhance robustness and efficiency through ensemble learning and optimization techniques. In practical applications, the appropriate model can be selected based on the specific characteristics and requirements of the task to achieve optimal classification performance.

## Hyparameter sensitivity analysis

This section analyzes the influence of key hyperparameter configurations on the performance of the ETX2Vec model. In particular, we systematically evaluate five core parameters associated with the temporal-biased random walk and embedding process: the temporal–amount weighting coefficient $\alpha$, the node embedding dimension $d$, the walk length $l$, the number of walks per node $r$, and the mapping interval ${[X}_{sup},{X}_{inf}]$ used for linear timestamp scaling. For each parameter, we examine how different settings affect the accuracy, precision, recall, F1 score, and mean performance of the downstream fraud classification task. This sensitivity analysis enables us to assess performance robustness and determine appropriate default configurations for ETX2Vec.

**Fig. 5:**
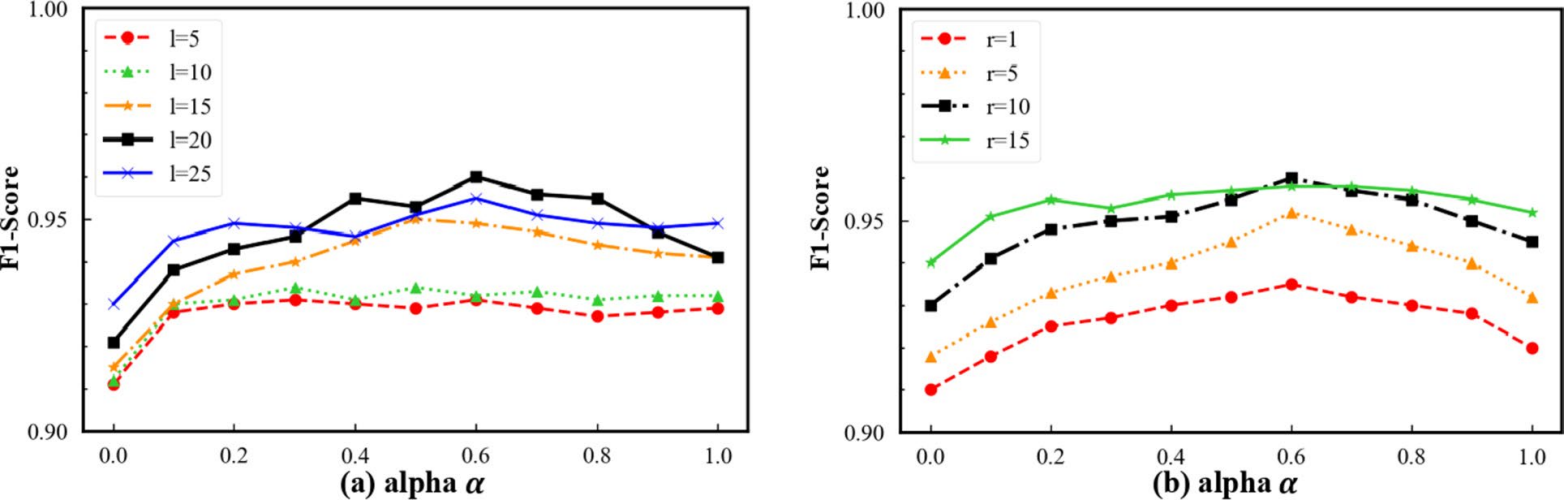
Joint effects of temporal weight $\boldsymbol{\alpha}$ with random walk context scale on model performance.

As shown in Fig. 5(a), we examine the variation of model performance with respect to the temporal weight coefficient $\alpha$ under different random walk lengths $l$. When $l$ is small (e.g., $l=5,10$), the overall performance is limited and exhibits weak sensitivity to $\alpha$, indicating that under insufficient contextual coverage, the temporal bias is unable to effectively capture cross-transaction temporal dependencies. As $l$ increases to 15 and 20, performance improves markedly and reaches its maximum around $\alpha=0.6$. This suggests that, for random walks of moderate length, an appropriate temporal constraint can suppress cross-time noise while preserving sufficient path diversity, thereby enabling the learning of more discriminative node representations. When $l$ is further increased, the performance gain saturates or even fluctuates, implying that excessively long walks dilute temporal consistency and weaken the benefit of temporal bias.

Figure 5(b) reports the performance trends under different numbers of walks per node $r$. A similar unimodal pattern with respect to $\alpha$ is observed, while the overall performance level increases substantially as $r$ grows. When $r$ is small, node embeddings are strongly affected by path stochasticity, preventing the temporal bias from being stably exploited. Increasing $r$ to 10 yields the best performance, with particularly stable behavior around $\alpha=0.6$, indicating that an appropriate sampling density allows temporally biased random walks to consistently reinforce coherent temporal patterns. Further increasing $r$ leads to marginal gains or slight degradation, suggesting that excessive path sampling may introduce redundant transaction patterns and impair generalization.

Taken together, these results demonstrate that the temporal weight $\alpha$ is not an independent hyperparameter; its effectiveness is tightly coupled with both the contextual length and the sampling density of random walks. Isolated single-parameter tuning is therefore insufficient to characterize the true performance boundary of the model, whereas joint optimization of the $\alpha-l$ and $\alpha-r$ parameter pairs is essential for fully exploiting the advantages of temporally biased random walks.

**Fig. 6:**
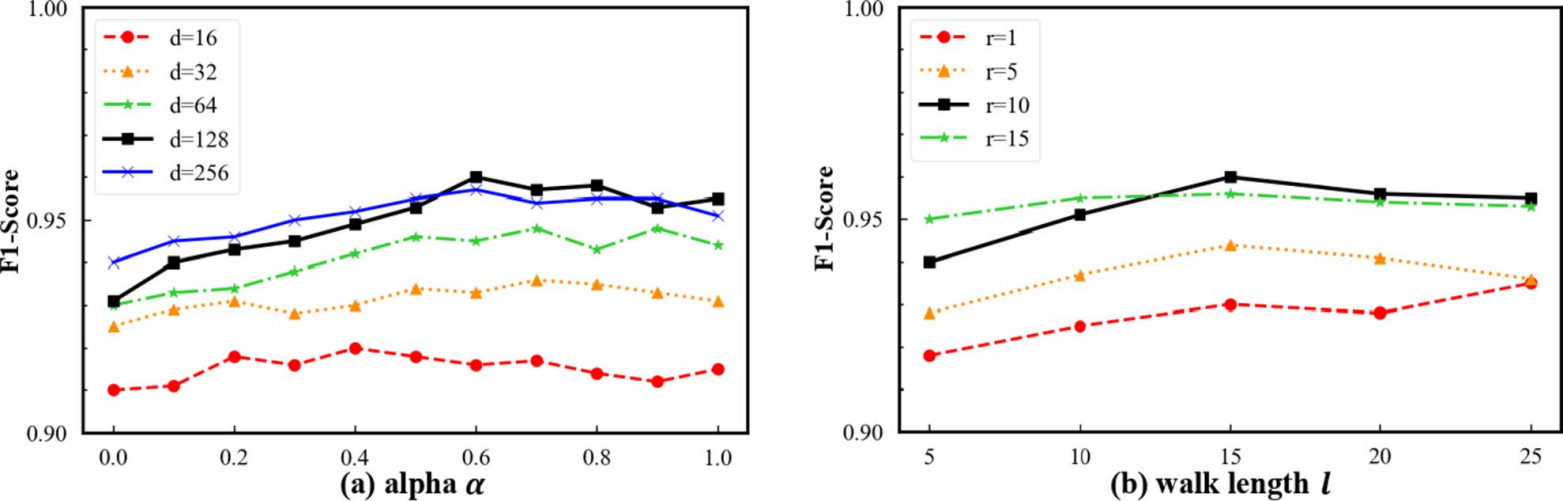
Synergistic effects of temporal bias, embedding capacity, and random walk depth.

We further investigate the synergistic effects between the temporal bias parameter $\alpha$ and the embedding dimension $d$, as well as the random walk depth parameters ($l$, $r$). As shown in Fig. 6(a), under different embedding dimensions, the model performance generally increases steadily with $\alpha$ and reaches its peak in the medium-to-high $\alpha$ range. This trend indicates a strong complementarity between temporally biased information and high-dimensional representation spaces: larger $d$ provides sufficient capacity to encode richer structural and temporal features, while the introduction of $\alpha$ effectively guides the model to focus on transaction paths with stronger causal and temporal consistency in the embedding space. Meanwhile, increasing $d$ from 16 to 128 leads to a substantial improvement in F1-score, whereas further expanding the dimension to 256 yields diminishing returns, suggesting the existence of a reasonable upper bound on embedding dimensionality for capturing higher-order structural information.

In conjunction with random walk parameter analysis, Fig. 6(b) shows that increasing the walk length $l$ and the number of walks per node $r$ consistently improves model performance, with particularly stable gains observed when $r\ge10$. However, performance tends to saturate when $r=15$, indicating that sufficiently dense path sampling facilitates the formation of more robust global structural representations during embedding learning, while excessive sampling provides limited additional benefit. Overall, these results validate a clear cooperative mechanism among $\alpha$, $d$, and $l$, $r$: the temporal bias performs structural filtering, random walks ensure effective information coverage, and the embedding dimension supplies adequate representational capacity. Together, they enable the model to achieve a well-balanced trade-off between performance and stability.

**Fig. 7:**
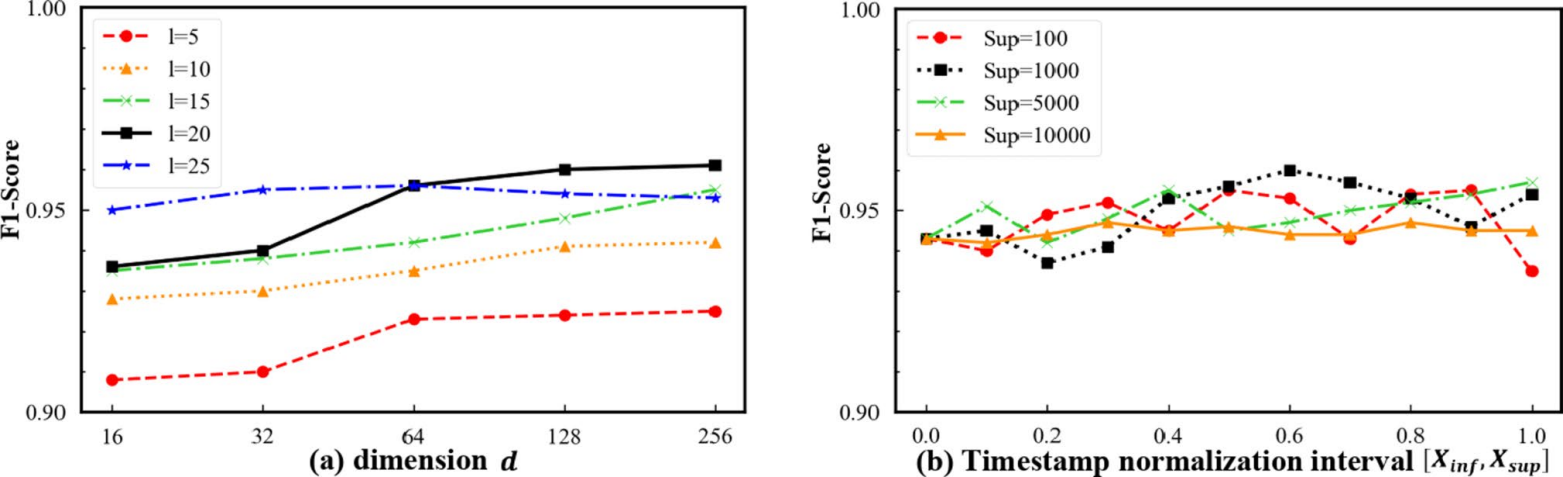
Coupled effects of embedding dimension and walk length, and robustness of temporal bias.

Finally, we examine the coupled effects between the embedding dimension $d$ and the random walk length $l$, as well as the stability of the temporal bias parameter $\alpha$ under different normalization ranges. As shown in Fig. 7(a), the joint $d-l$ results reveal a clear synergistic improvement trend as both $d$ and $l$ increase. When $l\le15$, enlarging $d$ yields only marginal performance gains; however, at $l=20$, the advantages of higher-dimensional embeddings ($d=128,256$) become substantially amplified. This observation indicates that moderately longer walk sequences provide sufficient structural context to fully exploit the representational capacity of high-dimensional embeddings. When $l$ is further increased to 25, performance gains saturate or even slightly decline, suggesting that excessively long paths may introduce redundant or noisy information that weakens effective representation learning.

On the other hand, Fig. 7(b) shows that under different normalization upper bounds, the performance curves exhibit mild fluctuations, while the optimal $\alpha$ consistently lies in the medium-to-high range (approximately 0.5–0.7), with only minor numerical variations. Notably, when the normalization range is set to [1, 1000], the model achieves its peak performance at $\alpha=0.6$. These results indicate that fixing the normalization range does not introduce structural bias into the model: the role of $\alpha$ primarily reflects a relative trade-off between temporal ordering and structural paths, rather than a sensitivity to absolute numerical scales. Moreover, the absence of sharp curve crossings or instability across different normalization bounds demonstrates strong robustness to normalization choices, providing solid justification for adopting a fixed and reproducible normalization strategy in practical deployment scenarios.

## Conclusions and future research

Graph embedding techniques map the topology and attributes of nodes and edges to a low-dimensional dense vector space, making them a mainstream approach for graph data analysis. Random walk-based graph embeddings, in particular, offer unique advantages in understanding Ethereum transaction networks. Unlike traditional information propagation networks, Ethereum’s nodes and edges not only carry rich transactional attributes but also encode complex trading patterns. However, traditional random walk strategies, while effective in capturing local and global structural information, fail to account for transaction-specific attributes such as timestamps and amounts. On the other hand, existing biased random walk strategies incorporate some transaction attributes but lack the ability to capture dynamic temporal sequences and capital flow patterns, which affects the quality of node embeddings and, consequently, the performance of downstream tasks.

In this context, building upon existing research, this paper proposes an Ethereum fraud detection algorithm, ETX2Vec, which incorporates a temporal-biased random walk strategy to better capture the complex characteristics of the Ethereum transaction network. The algorithm improves upon two key aspects: transaction subgraph construction and the random walk strategy.

First, transaction subgraphs are constructed by selecting the first-order predecessor and successor neighbor nodes. This approach not only enhances the similarity between the transaction subgraph and the original graph but also effectively captures the target node’s inbound and outbound transaction records. This step lays the foundation for capturing complete capital flow paths, which are critical for accurate fraud detection; Second, in terms of the random walk strategy, we introduce two key innovations. One approach ensures that node sequences reflect the dynamic temporal order of transactions by selecting the next node based on the non-decreasing principle of transaction timestamps. The other is a biased random walk strategy, where transaction timestamps and amounts influence the node transition probabilities. A parameter, α, is introduced to adjust the weight of these factors, enabling the model to capture both the dynamic sequence of transactions and large capital flow paths within the network. Experimental results show that ETX2Vec achieves an accuracy of 96.14%, precision of 95.58%, recall of 96.43%, F1 score of 96.00%, and an average performance of 96.04% in downstream node classification tasks. Compared to the best models in similar studies, ETX2Vec improves performance by 3.74%, even outperforming neural network models such as GAT and GCN. These results demonstrate that ETX2Vec is more effective in understanding and processing Ethereum transaction networks, ultimately leading to high-quality node embeddings and enhanced fraud detection capabilities.

The next steps of this research focus on three main directions: (1) integrating multi-source heterogeneous data—including graph-structured transaction data, basic transaction attributes, and external information such as user feedback, market trends, and news events—to obtain richer node representations; (2) extending the method to other blockchain platforms to assess its generalizability and robustness; (3) exploring near real-time or low-latency fraud detection by incorporating streaming or incremental embedding updates. While ETX2Vec currently operates in an offline setting to ensure high-quality embeddings, future enhancements in this direction could enable timely detection and risk assessment in rapidly evolving blockchain networks, bridging the gap between offline analysis and operational deployment.

## Data Availability

Datasets and source code are available from the GitHub repository: [https://github.com/Lujiarong1203/ETH2Vec](https:/github.com/Lujiarong1203/ETH2Vec) .
